# Current trends in urogynecological surgeries in Poland

**DOI:** 10.1007/s00192-019-04064-y

**Published:** 2019-07-31

**Authors:** Katarzyna Skorupska, Tomasz Rechberger, Michał Bogusiewicz, Aneta Adamiak-Godlewska, Agnieszka Kwiatkowska, Paweł Miotła

**Affiliations:** 2nd Department of Gynecology, Medical University in Lublin, ul. Jaczewskiego 8, 20-954 Lublin, Poland

**Keywords:** FDA safety communication, Prolapse surgery trends, Urinary incontinence surgery trends

## Abstract

**Introduction and hypothesis:**

Pelvic organ prolapse (POP) treatment has become more common in the world’s health care systems, and the demand for pelvic floor disorder rehabilitation has been projected to increase by 35% between 2010 and 2030. Restitution procedures vary, but after the US Food and Drug Administration (FDA) notifications, the global use of mesh in vaginal surgeries has significantly decreased. The aim of this study is to show trends in urogynecological surgeries in Poland.

**Methods:**

Retrospective analysis was performed of data obtained between 2009 and 2017 from the National Health Fund Information Centre website. Vaginal hysterectomies (VH), POP and urinary incontinence (UI) surgeries were considered.

**Results:**

In the study, 327,294 hospitalizations between 2009 and 2017 were considered: 29,821 VH, 265,147 POP and 53,328 UI procedures. Between 2009 and 2015, a rapid increase in the number of POP procedures was observed (r^2^ = 0.94, b = 1711, *p* < 0.001). The following years, however, were characterized by a marked decline in the number of POP surgeries. In addition, the number of vaginal suspensions with mesh dropped by 24.7%, posterior and anterior repair by 8.5%, and posterior repair by 7.5%, but the number of anterior repair procedures increased slightly by 1.5%. Moreover, between 2015 and 2017, the number of vaginal hysterectomies decreased by 9%. The number of UI surgeries had increased between 2011 and 2015 and then remained at a relatively stable level. A similar trend was observed for tape procedures, whereas the popularity of colposuspension has declined noticeably.

**Conclusions:**

The impact of FDA notifications has been observed in Poland as a decrease in TVM surgeries.

## Introduction

Due to an aging population, the need for treatment of pelvic organ prolapse (POP) has become a great challenge for medical professionals and for the public health care system in general. In Poland, 15.4% of the entire population is > 65 years old, and, as of 2017, life expectancy for women at birth is 81.8 years [1]. The demand for care of pelvic floor disorders has been projected to increase by 35% between 2010 and 2030 [2]. There are many surgical options for POP treatment–both native tissue and synthetic mesh operations. However, in January 2016, after previous notifications in 2008 and an update in 2011, the US Food and Drug Administration (FDA) reclassified surgical mesh for transvaginal repair of pelvic organ prolapse into class 3, which requires premarket approval (PMA) applications, and finally banned meshes in 2019 (https://www.fda.gov/MedicalDevices/ProductsandMedicalProcedures/ImplantsandProsthetics/UroGynSurgicalMesh/default.htm). In 2015, the Scientific Committee on Emerging and Newly Identified Health Risks (SCENIHR) recommended that any vaginal mesh usage should be considered only in complex cases following POP recurrence (https://ec.europa.eu/health/scientific_committees/emerging/docs/scenihr_o_049.pdf). Since then, the global use of mesh in vaginal surgeries has significantly decreased. Major pelvic floor disorders that require the use of mesh are: midurethral sling (MUS) procedures-tension-free vaginal tape (TVT) and transobturator tape (TOT) for stress urinary incontinence, transvaginal mesh (TVM) for POP and transabdominal mesh for POP. While the MUS procedure is the standard in stress urinary incontinence patients [3], in England this operation was paused after July 2018 till further safety measures are available (https://www.rcog.org.uk/en/patients/patient-leaflets/mid-urethal-sling-operation-for-stress-urinary-incontinence/). TVM surgery has been replaced by other procedures such as sacrocolpopexy or classical colporraphy [4].

Throughout the years, various approaches have been introduced to treat POP; still, the type of surgery may depend on several factors, such as type and degree of POP, patient characteristics and surgical expertise [5]. Other reasons include the practice pattern variation (PPV)—the difference in care that cannot be explained by the underlying medical condition. A Dutch study showed high PPV in the surgical treatment of POP and UI with respect to the choice of surgical treatment and the type of surgery [6]. Moreover, the authors of this study noted a high PPV per hospital and per region: some hospitals performed only hysterectomies for POP, while others used mostly uterus-preserving techniques. High PPV in TVM placement in different countries was also observed.

Since the global use of mesh in urogynecological procedures has significantly decreased, it is of great interest to know how this tendency affected the performance in urogynecological surgeries overall. The aim of this study was to investigate trends in POP and UI surgeries in Poland between 2009–2017.

## Materials and methods

A retrospective analysis of data from the public National Health Fund Information Centre websites was performed (https://prog.nfz.gov.pl/APP-JGP/KatalogJGP.aspx), and annual reports from 2009 to 2017 were analyzed. The database contains statistical information about the number of hospitalizations due to a specific disease per year and number of performed procedures in Poland. There is no information about the indication for those procedures. Reasons for hospitalization and type of procedures were identified using the ICD-9 classification. The same codes were used for each year of the study period, and the prevalence of pelvic organ prolapse, urinary incontinence and vaginal hysterectomies was investigated (Table 1). Subsequently, linear regression models were constructed for the observed trends. *P* < 0.05 was considered statistically significant.

**Table 1:** Pelvic organ prolapse (POP) and urinary incontinence (UI) procedures between 2009 and 2017 in Poland

Year	Total number of POP procedures	ICD-9					Total number of UI procedures	ICD-9	
		70.50	70.77	70.521	70.511	67.4		59.795 and 59. 791	59.44 and 59.51
2009	23,188	9802	6566	3367	1776	1677	2589	1416	1173
2010	25,551	10,312	8191	3527	1928	1593	2000	1049	951
2011	27,282	10,574	9180	3783	2061	1684	1808	988	820
2012	30,003	11,310	9831	4368	2531	1720	2639	1772	777
2013	31,690	12,264	10,514	4803	2611	1498	3378	2376	677
2014	30,686	12,218	9208	4768	2656	1545	4503	3464	497
2015	34,262	13,903	10,745	5065	2742	1467	5256	4159	500
2016	32,285	13,693	9326	4858	2545	1462	5288	4167	380
2017	30,200	12,817	8094	4710	2782	1377	4865	3746	345

70.50: repair of cystocele and rectocele, 70.77: vaginal suspension and fixation, 70.521: repair of rectocele, 70.511: repair of cystocele (anterior vaginoplasty with excision of urethral diverticulum), 67.4: amputation of cervix with vaginoplasty, 59.795: vaginal urinary incontinence surgery with tape, 59.791: anterior urethropexy, 59.51: Burch operation, 59.44: uretrocystopexy by suprapubic suspension

Ethics approval was not required because we used public domain and anonymous data.

## Results

**Fig. 1:**
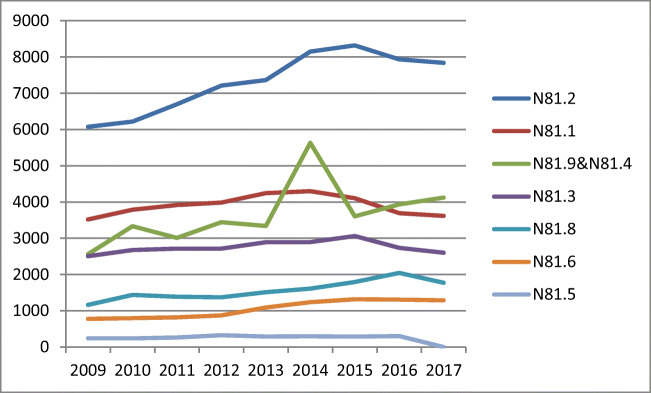
Number of hospitalizations due to POP between 2009 and 2017 in Poland. N81.2: incomplete uterovaginal prolapse, N81.1: cystocele, N81.9: female genital prolapse, unspecified, N81.4: uterovaginal prolapse, unspecified, N81.3: complete uterovaginal prolapse, N81.8: other female genital prolapse, N81.6: rectocele, N81.5: vaginal enterocele

The analysis comprised 327,294 hospitalizations between 2009 and 2017. Most (*n* = 265,147, 81%) were due to POP. The total number of POP procedures rose from 2009, had its peak in 2015 and decreased by 2017 (Fig. 3). Incomplete uterovaginal prolapse was the most common diagnosis (*n* = 65,803). Complete prolapse was diagnosed in 24,788 cases, and unspecified prolapse occurred in 32,984 patients. While 35,167 women had cystocele, rectocele was identified in 9508 patients. Enterocele was the least frequently diagnosed situation (*n* = 2236) (Fig. 1).

The most common procedure for POP was combined anterior and posterior colporrhaphy (*n* = 64,895). Suspension and fixation of the vagina using a mesh was performed in 47,887 patients; however, 24,204 women had posterior colporrhaphy and 13,336 anterior colporrhaphy. Finally, 7349 cervical amputations with simultaneous vaginoplasty for POP were performed. In addition, during the study period, 29,821 vaginal hysterectomies (VH) were undertaken.

Between 2009 and 2015, a rapid increase in the number of POP procedures was observed (r^2^ = 0.94, b = 1711, *p* < 0.001). The following years were, however, characterized by a marked decline in the number of POP surgeries (Fig. 1, Table 1). Prediction based on the trend between 2009 and 2015 revealed that in 2017 the number of procedures should have reached 37,505 [95% CI (34,775–40,234)], but only 30,200 were performed. In addition, between 2015 and 2017, the number of vaginal suspensions with mesh dropped by 24.7%, posterior and anterior repair by 8.5%, posterior repair by 7.5% and vaginoplasty with cervix removal by 6.5%. In contrast, the figure for the number of anterior repair procedures increased slightly by 1.5%. Moreover, between 2015 and 2017, the number of vaginal hysterectomies (performed for all indications) decreased by 9.0% (Fig. 5).

**Fig. 2:**
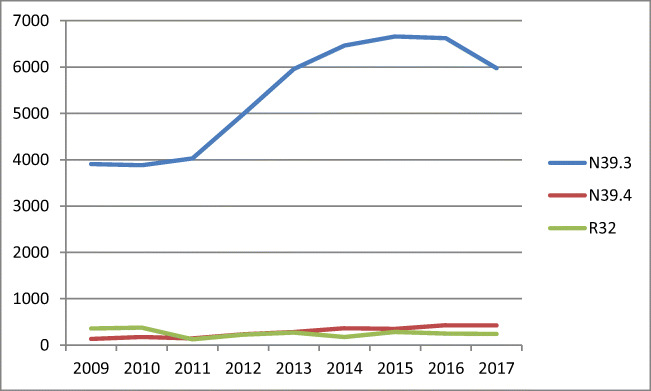
Number of hospitalization due to UI between 2009 and 2017 in Poland. N39.3: stress incontinence (female), N39.4: other specified urinary incontinence, R32: unspecified urinary incontinence

Among 53,328 hospitalizations for urinary incontinence (UI), 48,477 were due to stress urinary incontinence (SUI) and 4851 due to ‘other’ types (Fig. 2).

The number of hospitalizations and surgeries due to UI increased between 2011 and 2015 and then remained at a relatively stable level (Fig. 4). A similar trend was observed for tape procedures, whereas the popularity of colposuspension surgery had declined noticeably (r^2^ = −0.95, b = −99.1, *p* < 0.001) (Table 1). Vaginal urinary incontinence surgery with tape was the most common operation for urinary incontinence (*n* = 13921), Burch colposuspension occurred 2399 times, and vaginal surgery for SUI without tape was undertaken only 2014 times (*n* = 85). Furthermore, urinary incontinence surgery with the use of tape (sling) from the patient’s own tissues or synthetic material was performed in 405 cases.

**Fig. 3:**
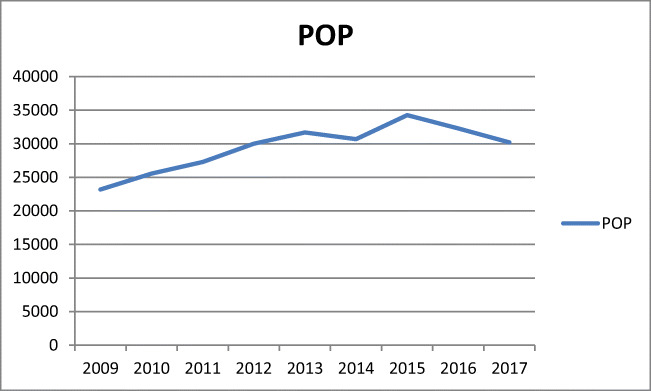
Total number of pelvic organ prolapse (POP) procedures between 2009 and 2017 in Poland

**Fig. 4:**
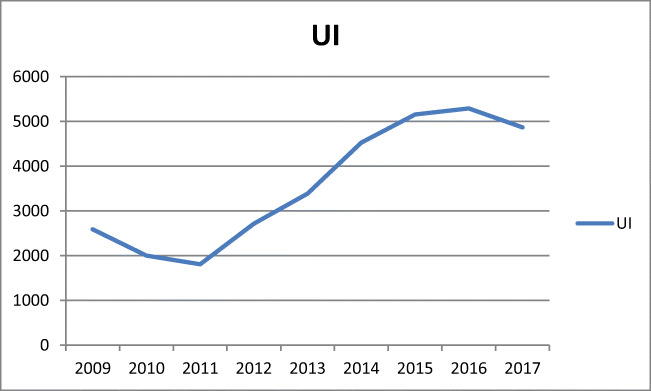
Total number of urinary incontinence (UI) procedures between 2009 and 2017 in Poland

Since 2015, a trend towards a decrease in the number of POP surgeries has been noticeable (Fig. 3). Although it has been a general tendency, the most marked decline was observed in mesh surgeries. Still, the number of UI procedures overall and tape procedures has remained at a similar level since 2015 (Fig. 4), while colposuspension has been an increasingly rare procedure.

## Discussion

The changes in surgical practice reported in Europe and the USA have also been reported in Poland [1]. We observed a decrease in POP surgeries with transvaginal mesh (TVM) in 2017 compared with previous years. Such surgeries peaked in 2015 in Poland; however, in other European countries, the number of TVM surgeries performed decreased earlier. In England, for example, colporrhaphy with mesh augmentation increased only initially after its introduction in 2007–2008, when 4.5% procedures were carried out, to peak at 5.6% in 2008–2009, followed by a constant decrease—with the lowest point of 1.5% seen in 2015–2016. Specifically, vaginal meshes have been mainly introduced to augment the anterior vaginal wall, with the number of performed procedures rising from 704 (2007–2008) to 908 (2008–2009) and then dropping to 231 (2015–2016) [7]. The Portuguese National Medical Registry also showed an increase in TVM surgery between 2007 and 2011—48% of all surgical mesh procedures were anterior vaginal wall operations. In 2012, however, although the use of vaginal mesh for apical defects almost doubled, the numbers for the anterior/posterior compartment showed a slight decrease [8]. In the USA, a significant decrease in TVM surgeries began in 2009 after the first FDA notification and continued after an update in 2011. Vaginal mesh procedures have declined over time (*p* = 0.001), comprising 27% of all repairs in early 2008, 15% at the first FDA notification, 5% by the FDA update and 2% at the end of 2011 [9]. As of April 16, 2019, the FDA banned the sale and distribution of transvaginal mesh for anterior prolapse repair (https://www.fda.gov/MedicalDevices/ProductsandMedicalProcedures/ImplantsandProsthetics/UroGynSurgicalMesh/default.htm). This will result in cessation of such surgeries. The OECD report also shows a decrease in the rate of transvaginal grafts per 1000 women between 2010 and 2012 in the USA, Canada and Germany and an increase in such procedures in Sweden, Israel, Holland and Denmark—the median being 3.74% [10]. Unfortunately, Poland was not included in the analysis.

The Polish National Health Fund Information Centre website (https://prog.nfz.gov.pl/APP-JGP/KatalogJGP.aspx) does not provide information about apical defects, whereas this information is available in other countries databases, so we were not able to show how many surgeries were preformed as sacrocolpopexy or TVM because of this condition. In the OECD report, however, the rate of sacral colpopexy per 1000 women between 2010 and 2012 increased in most countries (Germany, Ireland, the USA, Holland, Sweden, Australia, France, Canada) and decreased only in Denmark–the median being 25.55 [9]. Of note, Polish national statistics distinguish total and partial prolapse and bladder and rectal divergence—understood by us as cystocele and rectocele. Moreover, between 2009 and 2016, the database also showed information about enterocele, but this information was no longer available in 2017.

In Poland, the number of anterior and posterior colporraphies performed did not change between 2012 and 2017 and remained at a similar level in the recent years. These procedures were the most common operations due to POP in Poland, England, Portugal and the USA. In other European countries, however, a decrease in the number of such procedures has been noted. England’s statistics indicated a rise in colporraphies from 2005 to 2010, followed by a plateau until 2014 and a subsequent negative trend in 2015 and 2016 [7]. The number of native tissue anterior/posterior repairs in Portugal (repair of cystocele and/or rectocele) increased significantly over time, comprising 1140 repairs in 2000 and 3280 in 2012. Thus, native tissue anterior/posterior repair was the most common type of surgery—with 30,169 cases [7]. In the USA, the percentage of native tissue anterior/posterior repairs (*p* < 0.001) and apical suspensions (*p* = 0.007) increased with time, whereas colpocleisis remained constant (*p* = 0.475) between 2008 and 2011 [9].

**Fig. 5:**
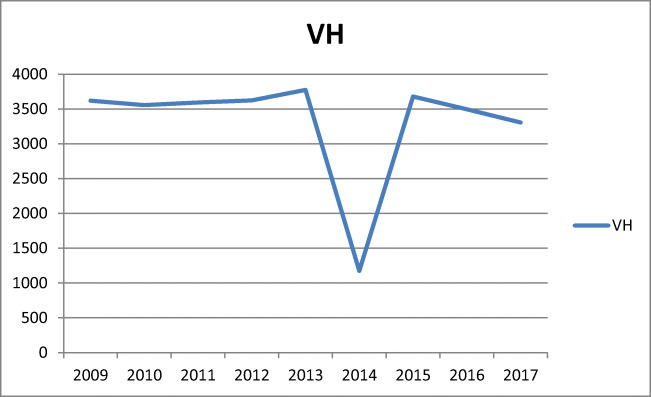
Total number of vaginal hysterectomies between 2009 and 2017 in Poland. *Lack of sufficient data in 2014

The number of vaginal hysterectomies in Poland during the study period was at the same level between 2009 and 2013, decreased between 2013 and 2014 and then increased between 2014 and 2015, to decrease again from 2015 to 2017 (Fig. 5). The data from year 2014 are not complete in the database—there is a lack of information from this period of time; hence, it was excluded it from evaluation. VH is one of the techniques for apical defect correction. A Danish study showed a decrease in the number of VHs and an increase in the number of uterus-preserving operations performed from 2010 to 2016 [10].

Although stress urinary incontinence (SUI) surgery evolved significantly after introducing synthetic MUS, the worldwide performance of these operations has also been affected by the FDA warning. The OECD report shows a decrease in the rate of MUS per 1000 women between 2010 and 2012 in the USA, Denmark, Canada, France and Australia and an increase in Sweden and Holland—the median being 3.97 [11]. In England, a rise in the number of sling surgeries was noted until 2008–2009, followed by a consistent drop, with a nadir of 6383 procedures in 2016–2017. Furthermore, traditional operations such as colposuspensions markedly decreased to 189 in 2012–2013 [12]. Different trends were observed in Canada between 2006 and 2011—no change in the number of performed sling surgeries was indicated. Hence, the conclusion was that either the FDA notification had little influence on local surgery practices or that safety was already at such a high level that improvements were not possible [13]. Analysis of the Polish database showed an increase in UI procedures between 2010 and 2016 and a slight decrease from 2016 in the number of sling surgeries and Burch colposuspensions performed (https://prog.nfz.gov.pl/APP-JGP/KatalogJGP.aspx). Furthermore, the number of colposuspensions decreased markedly throughout the study period—probably due to the increased number of MUS surgeries and the fact that surgeons are not familiar with traditional SUI operations. There was also a decrease in the number of hospitalizations due to SUI and other types of UI.

The limitations of this study are that the Polish database does not distinguish between indications for VH so we were not able to state how many of these were due to prolapse and that the database does not provide information about apical defects and procedures performed because of this condition.

## Conclusion

The impact of the FDA notifications has been seen in Poland as a decrease in mesh and artificial tape procedures. Unfortunately, the Polish database does not distinguish between the apical type of prolapse and sacrocolpopexy so it was not possible to show the number of such procedures undertaken. Another limitation is that we cannot differentiate between primary and recurrent surgeries.
